# Health of migrants and other vulnerable populations across Asian countries: Build Forward Better beyond the COVID-19 pandemic

**DOI:** 10.1186/s41182-023-00543-7

**Published:** 2023-09-11

**Authors:** Azusa Iwamoto, Masami Fujita, Dang Quang Luong, Jongsoh Anh, Betty Ya-Wen Chiu, Jaewook Choi, Hung-Yi Chiou, Reiko Hayashi, Jun Kobayashi

**Affiliations:** 1 Bureau of International Cooperation, National Center for Global Health and Medicine, 1-21-1, Toyama, Shinjuku-Ku, Tokyo, 162-8655 Japan; 2 Personnel Department, General Office for Population and Family Planning, Ministry of Health, Secretariat of Migrant Health Working Group, Hanoi, Vietnam; 3 Humanitarian Development and Migrant Health Team, Global Cooperation Center, Korea Foundation for International Healthcare, Soul, Republic of Korea; 4 Centre for Global Health and Security, Department of Public Health, Kaohsiung Medical University, Kaohsiung, Taiwan; 5 Department of Preventive Medicine, Korea University, Soul, Republic of Korea; 6 Institute of Population Health Sciences, National Health Research Institutes, Miaoli, Taiwan; 7 National Institute of Population and Social Security Research, Tokyo, Japan; 8 Graduate School of Health Sciences, University of the Ryukyus, Okinawa, Japan

**Keywords:** Asia, Health security, Migration and health, Network, Public health emergency, Vulnerable populations, Integration

## Abstract

Global migration has been increasing since before the COVID-19 pandemic. The pandemic has clearly shown a lack of preparedness for the next public health emergency when it comes to vulnerable populations including migrants. To include the issues of migration and health in the current global health agenda, it is important to establish/strengthen a network for collaboration among various stakeholders from both the migrant-sending and host countries of migrants especially in the Asian-Pacific region. As the initial step for networking in Asia, in March 2023, a hybrid style international symposium was held in Japan and agreed on a goal and five pillars: surveillance and monitoring, risk communications, community engagement, access to health and social protection services, and supportive environments. Considering the transition of context from the COVID-19 crisis to ‘Build Forward Better’, through the Asian network, we will envisage the better world, where vulnerable populations including migrants will not be left behind from health security.

## Background

As migrant populations in the world have been increasing since before the COVID-19 pandemic [1], international migration has become an important global agenda in the twenty-first century. In May 2022, the 1st International Migration Review Forum (IMRF) was held in New York 4 years after the adoption of the Global Compact for Migration (GCM). United Nations organizations, including the World Health Organization (WHO) and International Organization for Migration (IOM) have been challenging the health aspects of this issue. For instance, the WHO published the first world report on the health of refugees and migrants in 2022 [2]. However, we are still at the initial stages of its agenda.

Around the world, there have been various public health emergencies, including pandemics, natural disasters, climate crises, conflicts and wars. For example, over eight million Ukrainians need health supports in European countries because they have evacuated from their home country because of the war [3]. For such vulnerable populations (VPs), emergency responses should be urgently arranged to provide essential health services such as vaccination, emergency medical, surgical and obstetric and neonatal care, and primary care for communicable and non-communicable diseases, in receiving countries [3]. The COVID-19 pandemic has clearly shown a lack of preparedness for the next public health emergency when it comes to VPs including migrants, refugees, people experiencing homelessness or living in slums, people with disabilities, people in poverty, people affected by the digital divide, people who are not protected from discrimination and exclusion by laws, policies and practices, and other vulnerable groups [4]. According to the global health security index 2021, only 33 of 195 countries had plans for emergency preparedness and a response for VPs including migrants [5]. This means that migrants have been continuously neglected.

To include the issues of migration and health in the current global health agenda, it is important to establish/strengthen a network for collaboration among various stakeholders from both the migrant-sending and host countries of migrants. In particular, in the Asian-Pacific region, networking on migration and health, with the inclusion of academia, nongovernmental organizations, civil societies, and governmental officials—as has been established in Europe and the Americas—is urgently needed [6, 7]. Furthermore, a multisectoral approach is crucial for this agenda because it is affected by various policies of not only the health sector, but also labor, industry, economy, immigration services, and other sectors.

### Symposium and consultation workshop in March 2023

As the first step for networking in Asia, on March 5, 2023, we held a hybrid style international symposium as part of the 41st Western Regional Conference of Japan Association for Global Health (JAGH) in Japan. The objectives of the symposium were (1) to share experiences/lessons learned from existing networks in Asian countries and (2) to consider the establishment of a network in Asia, with a new commitment to migration and health.

After explanation of the objectives by chairpersons, the following four presentations were held:

1. Dr. Masami Fujita (Migrant Neighbors’ Network & Action (MINNA)/National Center for Global Health and Medicine, Japan) highlighted the importance of integrating migrants and other VPs in the public health emergency preparedness.
2. Mr. Luong Quang Dang (Ministry of Health/Secretariat of the Migrant Health Working Group, Vietnam) introduced the experience of a multisectoral approach through the migrant health working group headed by the Ministry of Health in Vietnam.
3. Mr. Jongsoh Ahn (Korea Foundation for International Healthcare) explained their experiences and lessons learned from the viewpoint of health security mainly for migrant workers in the Republic of Korea on access to health services and registrations.
4. Dr. Jun Kobayashi (University of the Ryukyus) shared the lessons learned from an existing school health network promoted by the Japan Consortium for Global School Health Research (JC-GSHR) since 2010 to explain the needs of regional academic networks for the effective promotion of health programs.

The day before the symposium, we also conducted a consultation workshop to learn from each other and freely exchange ideas in relation to our expectations for the network in Asia, including future plans. Fifteen participants from Korea, Japan, Taiwan and Vietnam joined online.

Both the symposium and workshop were organized by two committees (Migration & Health and Global Networking) of JAGH and co-organized by the School of Tropical Medicine and Global Health, Nagasaki University, and MINNA.

### The way forward

At the end of the symposium, participants agreed to launch a network to work on the following five pillars to integrate VPs into ‘PPRR (prevention, preparedness, response, and recovery)’ plans for future public health emergencies:

1. Adequate understanding of VP communities through surveillance and monitoring,
2. Information dissemination and obtaining feedback by adequate risk communications with VPs to mitigate barriers to language, culture systems, etc.,
3. Involvement of VPs to solve problems associated with living together (community engagement),
4. Better access to health and social protection services for universal health coverage and universal social protection,
5. Creating broader supportive environments around VPs (considering social determinants of health, climate justice).

These pillars have been identified based on several global and regional guidance [4, 8, 9]. An interim guidance of WHO Western Pacific Regional Office (WPRO) underlined the importance of inclusion of VPs into routine and improved surveillance systems [4]. The guidance indicated practical actions such as mapping of VPs including social determinant factors, accurate data collection in collaboration with reliable community leaders beyond the health sector, and dissemination of user-friendly information [4], in which human rights-based approach should be incorporated [4, 8]. A working group consisting of several international organizations in Asia identified steps to strengthen risk communications and community engagement (RCCE) for VPs, which include collaborating with VP community network to monitor risks, diversifying communication tools using simplifying messages, establishing continued feedback mechanisms for modification and adjustment, among others [9]. Concerning access to health services, the WHO WPRO developed an interim guidance to protect and care VPs amid the COVID-19 pandemic, which articulated common principles for a wide range of VPs and specific practical considerations for each VP such as migrants [8].

The network plans to conduct a series of activities. To begin with, taskforces on surveillance, RCCE and access to health services have been established and are currently reviewing global literature to identify the progress and challenges of relevant subjects. They will assess the extent of integration of migrants and other VPs in health systems of respective countries, pool the experiences of measures for operationalizing the integration, further engage those involved in policy and system development, implementation, and research. They will also organize discussions to identify challenges and possible solutions for integrating migrants in future pandemic preparedness.

Taking into account the transition of context from the COVID-19 crisis to ‘Build Forward Better’ after the pandemic, we wish to mainstream the health of migrants and other VPs in the global agenda, from health security to universal health coverage including social health protection. ‘Building forward’ means ‘people who tend to be left behind are actively encouraged and supported to be in the front, engaging in informed and meaningful participation in the decision-making processes that directly affect their lives’ [10]. This means that it is crucially important not to revive the same conditions that existed before the COVID-19 pandemic but to advance to more inclusive health governance. By creating a network of academia, public institutions and civil societies between migrant-receiving and sending countries in Asia, we believe that it will be possible to not only create a movement from the bottom up but also provide the necessary scientific evidence for governments to make policy decisions. For better evidence-based interventions, the future research agenda we currently consider important include practical surveillance systems minimizing negative impacts on human rights, effective communication channels, equitable entitlements for health services, and collaboration between health services and social protection schemes. We plan to review and adjust these agenda through consultations with a wider range of stakeholders as per evolution of our initiatives.

We hope our network works for actualization of the world, where VPs—including migrants—will not be left behind from health security and where universal health care and universal social protection will be achieved in the future.

## Data Availability

Not applicable.
